# Associations between conduct problems in childhood and adverse outcomes in emerging adulthood: a longitudinal Swedish nationwide twin cohort

**DOI:** 10.1111/jcpp.13169

**Published:** 2019-12-18

**Authors:** Paul Lichtenstein, Martin Cederlöf, Sebastian Lundström, Brian M. D'Onofrio, Henrik Anckarsäter, Henrik Larsson, Erik Pettersson

**Affiliations:** ^1^ Department of Medical Epidemiology and Biostatistics Karolinska Institutet Stockholm Sweden; ^2^ Centre for Ethics, Law and Mental Health (CELAM) University of Gothenburg Gothenburg Sweden; ^3^ Gillberg Neuropsychiatry Centre University of Gothenburg Gothenburg Sweden; ^4^ Department of Psychological and Brain Sciences Indiana University Bloomington IN USA; ^5^ School of Medical Sciences Örebro University Örebro Sweden

**Keywords:** Twins, comorbidity, externalizing disorder, internalizing disorder, conduct disorder

## Abstract

**Background:**

We examined whether childhood conduct problems predicted a wide range of adverse outcomes in emerging adulthood and whether the association with internalizing problems remained after adjusting for general comorbidity and externalizing problems.

**Methods:**

Participants were 18,649 twins from the Child and Adolescent Twin Study in Sweden. At age 9/12, parents rated their children on eight conduct problems. Adverse outcomes were retrieved from national registers in emerging adulthood (median follow‐up time = 9.2 years), including diagnoses of six psychiatric disorders, prescriptions of antidepressants, suicide attempts, criminality, high school ineligibility, and social welfare recipiency. We estimated risk for the separate outcomes and examined if conduct problems predicted an internalizing factor above and beyond a general comorbidity and an externalizing factor. We used twin analyses to estimate genetic and environmental contributions to these associations.

**Results:**

On the average, each additional conduct symptom in childhood was associated with a 32% increased risk of the adverse outcomes in emerging adulthood (mean hazard ratio = 1.32; range = 1.16, 1.56). A latent childhood conduct problems factor predicted the internalizing factor in emerging adulthood (β_boys_ = .24, standard error, *SE* = 0.03; β_girls_ = .17, *SE* = 0.03), above and beyond its association with the externalizing (β_boys_ = 0.21, *SE* = 0.04; β_girls_ = 0.17, *SE* = 0.05) and general factors (β_boys_ = 0.45, *SE* = 0.03; β_girls_ = 0.34, *SE* = 0.04). These associations were differentially influenced by genetic and environmental factors.

**Conclusions:**

It is important to monitor boys and girls with conduct problems not only for future externalizing problems, but also for future internalizing problems. Prevention of specific outcomes, however, might require interventions at different levels.

## Introduction

Conduct problems are one of the most prevalent forms of maladjustment in childhood (Erskine et al., 2014). Longitudinal studies have shown that childhood conduct problems are associated with subsequent externalizing problems such as criminality and substance abuse (Erskine et al., 2014; Fergusson, Horwood, & Ridder, 2005). Some studies have also shown that children with conduct problems are at elevated risk of developing internalizing problems such as depression and anxiety, in addition to numerous nonpsychiatric adversities, including educational problems, unemployment, and poor health outcomes (Caspi, Wright, Moffitt, & Silva, 1998; Deffenbacher, Deffenbacher, Lynch, & Richards, 2003; Fergusson et al., 2005; Kim‐Cohen et al., 2003; Tremblay et al., 1992).

Although childhood conduct problems appear to be associated with a host of subsequent problems, the generalizability of past research efforts is somewhat limited. First, most past studies are based on relatively small samples, including two birth cohorts in New Zeeland (*N* = 1,265 and 1,037, respectively; Fergusson et al., 2005; Kim‐Cohen et al., 2003). Small samples generate less accurate risk estimates, particularly when outcomes are rare. Furthermore, it is challenging to split smaller samples by sex, even though conduct problems in childhood and its sequelae in emerging adulthood might differ between boys and girls (Archer, 2004; Bettencourt & Miller, 1996; Eagly & Steffen, 1986). Second, outcomes have often been assessed with self‐reports, which are subject to potential biases (Takayanagi et al., 2014), and the most troubled individuals might be less likely to participate at follow‐up assessments. Relying on outcomes ascertained from population‐based registers partly remedies such problems. Third, the associations between childhood conduct problems and subsequent internalizing problems could occur because internalizing problems are correlated with externalizing problems (Caspi & Moffitt, 2018; Kendler et al., 2011; Krueger & Markon, 2006; Lahey, Krueger, Rathouz, Waldman, & Zald, 2017). Fourth, it remains somewhat uncertain how genes and the environment contribute to the longitudinal associations. This is important because a genetic origin implies that the association stems from internal factors, whereas an environmental origin points toward external factors.

We used a large, nationwide sample of Swedish twins to examine whether parent‐reported conduct problems in childhood was associated with register‐based externalizing and internalizing outcomes in emerging adulthood in men and women. We then assessed whether the association between childhood conduct problems and later internalizing problems remained after adjusting for general comorbidity and externalizing problems. In addition, we examined the genetic and environmental contributions to the observed associations.

## Methods

### Participants

Participants were 18,649 twins (49.0% females) born between 1992 and 2002 who had participated in the Child and Adolescent Twin Study in Sweden (CATSS), which is an ongoing longitudinal study targeting all twins born in Sweden since July 1, 1992 (Anckarsater et al., 2011). When the twins turned 9 or 12 years (CATSS‐9/12), their parents were phone‐interviewed about their health and behavior, including conduct problems. The response rate was 71%.

Analyses of differences between nonresponders and responders in the CATSS‐9/12 study were conducted on the first 11,222 participants, for which psychiatric and socioeconomic information was retrieved from national registers. Nonresponding parents were more likely to have been treated in psychiatric settings (9.6% vs. 6.3%), been convicted of a felony (6.4% vs. 4.0%), be divorced (16.4% vs. 12.4%), and to have a lower socioeconomic status (26.6% vs. 21.9%). Moreover, children of nonresponding parents had a higher prevalence of psychiatric diagnoses such as ADHD (2.1% vs. 1.6%), or autism spectrum disorders (0.95% vs. 0.84%), and 1.8% versus 1.4% had been prescribed with psychotropic treatment for ADHD (Anckarsater et al., 2011). To adjust for this unequal probability of nonresponse, we combined the four parental predictors of nonresponse into a binary category, and the child predictors of nonresponse into a binary category. Thus, there were four sets of response weights (i.e., parents and children with adverse outcomes; parents but not children with adverse outcomes; children but not parents with adverse outcomes; and neither parents nor children with adverse outcomes). We incorporated these response weights in the factor analyses, structural equation models, and twin analyses.

After receiving a complete description of the study, parents provided written informed consent on behalf of their children. This study was approved by the Regional Ethics Review Board in Stockholm.

### Exposure: childhood conduct problems

Parents were interviewed about their children’s mental health with the Autism‐Tics, ADHD and Other Comorbidities inventory (A‐TAC; Hansson et al., 2005; Larson et al., 2010), which includes measures of clinical features and diagnostic criteria of all major disorders in child‐ and adolescent psychiatry. All questions denoted a lifetime perspective, and the response alternatives were ‘no’, ‘yes, to some extent’, and ‘yes’. In the current study, conduct problems were measured with eight symptoms of oppositional defiant disorder (ODD; five symptoms) and conduct disorder (CD; three symptoms). See Table S1 for the complete set of symptoms. The area under the curve for ODD and CD was 0.80 and 0.70, respectively (Marland et al., 2017). See Table S2 for the prevalence of the number of children with conduct symptoms by sex.

### Outcomes: adverse events in emerging adulthood

The interview data were linked to several national registers. The National Patient Register (NPR) includes psychiatric inpatient and outpatient episodes. Diagnoses were assigned by the attending physician in a nonhierarchical manner, according to the tenth version of the International Classification of Diseases (World Health Organization, 1992). Validation studies have shown that psychiatric diagnoses in the NPR are generally accurate (Lichtenstein et al., 2006; Ludvigsson et al., 2011; Ruck et al., 2015; Sellgren, Landen, Lichtenstein, Hultman, & Langstrom, 2011). We included diagnoses of depression (F32–39), anxiety (F40–42, F44–45 or F48), suicide attempts (X60–84, or Y10–34), psychotic/bipolar disorder (F20–31), drug abuse (F11–19, except F11.5, F12.4, F12.5, F13.5, F14.5, F15.5, F16.5, F17.5, F18.5, and F19.5), and alcohol misuse (F10, except F10.5). The National Crime Register (NCR) contains records of all criminal convictions in district courts, and the Register of Persons Suspected of Offenses contains records of suspected crimes based on completed investigation by police, customs authority, or the prosecution service. From these registers, we extracted violent and nonviolent suspected or convicted crimes. The Prescribed Drug Register (PDR) contains information about dispensed medications, from where we extracted antidepressant/sedative medication prescription (Anatomical Therapeutic Chemical Classification; N05B‐C or N06A). We extracted information about ineligibility to begin high school at the end of the nine‐year compulsory primary school, which can occur due to low grades or excess absences, from the Primary School Register. We extracted information about social welfare recipiency from the Longitudinal Integration Database for Health Insurance and Labor Market Studies Register. See Table S2 for the prevalence of each outcome among individuals aged 17–22 years old.

### Statistical analyses

#### Cox regressions

First, we created a sum score variable by adding the eight conduct symptoms together (participants with a ‘yes’ or ‘yes, to some extent’ received a score of 1 on each symptom). We then regressed the adverse outcomes on the sum score using Cox regression with the end of coverage of the registers as censoring time (between December 31, 2013, and December 31, 2015). Time‐to‐event was calculated from the date participants completed the CATSS‐9/12 assessment. All analyses were conducted separately for men and women, and adjusted for the mothers' education level as a proxy for socioeconomic status.

#### Factor analyses of childhood conduct problems and adverse events in emerging adulthood

Second, we used confirmatory factor analysis (CFA) to extract a single factor from the eight childhood conduct problems. We applied exploratory factor analysis (EFA) to the eleven adverse outcomes. We applied EFA rather than CFA to the outcomes because we did not have an a priori hypothesis regarding their correlational structure, and because we anticipated that the data would not have simple structure. We determined how many exploratory factors to extract based on the scree plot, which involves identifying an ‘elbow’ in the plot of the eigenvalues against the eigenvectors (Cattell, 1966). As a sensitivity analysis, to ensure that the EFA loading pattern did not hinge on inclusion of less typical adverse outcomes including antidepressant medication prescription, high school ineligibility, and social welfare recipiency, we reran the EFA after excluding these variables. We then used the Direct Schmid–Leiman transformation to rotate the overlap among the extracted factors into a single general comorbidity factor, as well as several independent factors that captured specific covariation among subsets of outcomes not accounted for by the general factor (Waller, 2018). Although this rotation generates an extra factor above and beyond the extracted number of dimensions, the additional general factor is merely a reparameterization of the factor correlations. The factor rotation was conducted in R (R Core Team, 2017). Because both the conduct symptoms and the outcomes were skewed, we treated the data as categorical using the mean‐ and variance‐adjusted weighted least square estimator (WLSMV) in the Mplus software (Muthén & Muthén, 1998–2015).

#### General and specific factors regressed on the conduct problems factor

Third, we regressed the general and specific factors based on the adverse outcomes onto the latent conduct problems factor. This way, we could examine whether childhood conduct problems predicted later internalizing problems, above and beyond its associations with the general and externalizing factors. We stratified all models by sex, and statistically adjusted for year of birth.

Because the factor analysis and structural equation modeling (SEM) cannot take time‐to‐event into account (in contrast to the Cox analysis), for those analyses, we only included participants who were born between 1992 and 1997, such that they were between 17 and 22 years old at follow‐up, to ensure that they had lived long enough to risk suffering the adverse outcomes (*n* = 3,856 males; 3,508 females).

#### Twin analyses

Fourth, we decomposed the observed (phenotypic) regressions into that which could be attributed to genetics (additive effects of different alleles; β_a_); the shared environment (effect of growing up in the same household after controlling for genetics; β_c_); and the nonshared environment (environmental influences that make MZ twins within a pair different from one another*; *β_e_). For the observed outcomes, this corresponded to decomposing probit betas, as standard twin methods do not allow for decomposing bivariate time‐to‐event data (i.e., hazard ratios). See Appendix S1 for more detailed information.

If β_e_ is positive and significant, it could indicate that treatment of conduct problems at the individual level might prevent later outcomes (D'Onofrio, Lahey, Turkheimer, & Lichtenstein, 2013; Lahey & D'Onofrio, 2010; Turkheimer & Harden, 2014). However, this parameter also captures common causes unique to only one twin within a pair. For example, an early head injury to only one twin might increase the probability of childhood conduct problems and later adverse outcomes. In such instances, treatment of early conduct problems would not be expected to alter the probability of later adverse outcomes because the true origin was the nonshared head injury.

## Results

### Associations between childhood conduct symptom count and the adverse outcomes

Table 1 shows frequencies and proportions of individuals’ number of childhood conduct symptoms by the subsequent outcomes. Of 18,649 participants, between one and 120 individuals (depending on the outcome) were excluded as they had experienced the outcome before the baseline assessment. The median follow‐up time was 9.2 years, with the first quartile at 7.4 years and the third quartile at 10.2 years.

**Table 1:** Frequencies and proportions of individuals with and without childhood conduct symptoms by adverse outcomes in emerging adulthood by sex

	Childhood conduct symptoms					
	0	1	2	3	4	5–8
	*N* (%)	*N* (%)	*N* (%)	*N* (%)	*N* (%)	*N* (%)
Depression	198 (1.59)	60 (2.05)	42 (2.86)	27 (3.18)	13 (2.90)	22 (4.91)
Females	142 (2.21)	40 (2.97)	22 (3.31)	13 (3.85)	7 (3.98)	10 (6.58)
Males	56 (0.93)	20 (1.27)	20 (2.49)	14 (2.73)	6 (2.21)	12 (4.05)
Anxiety	253 (2.04)	59 (2.02)	55 (3.75)	46 (5.42)	20 (4.49)	30 (6.73)
Females	168 (2.61)	28 (2.08)	33 (4.97)	25 (7.42)	9 (5.17)	15 (10.00)
Males	85 (1.62)	31 (1.96)	22 (2.74)	21 (4.10)	11 (4.06)	15 (5.07)
Anti‐depressant/sedative medication	862 (6.95)	263 (9.05)	166 (11.41)	128 (15.27)	74 (16.70)	111 (25.69)
Females	544 (8.77)	145 (10.85)	80 (12.14)	55 (16.47)	28 (16.09)	40 (27.59)
Males	318 (5.32)	118 (7.51)	86 (10.8)	73 (14.43)	46 (17.10)	71 (24.74)
Suicide attempt	120 (0.97)	34 (1.17)	21 (1.44)	12 (1.42)	7 (1.57)	9 (2.03)
Females	67 (1.05)	17 (1.27)	11 (1.66)	6 (1.78)	3 (1.70)	6 (3,97)
Males	53 (0.89)	17 (1.08)	10 (1.25)	6 (1.17)	4 (1.48)	3 (1.03)
Severe mental illness	23 (0.18)	6 (0.21)	6 (0.41)	7 (0.82)	4 (0.89)	6 (1.35)
Females	15 (0.23)	3 (0.22)	3 (0.45)	4 (1.18)	1 (0.57)	3 (0.57)
Males	8 (0.13)	3 (0.19)	3 (0.37)	3 (0.58)	3 (1.10)	3 (1.97)
Substance abuse	44 (0.35)	13 (0.44)	16 (1.09)	5 (0.59)	5 (1.12)	7 (1.57)
Females	20 (0.31)	6 (0.45)	5 (0.75)	2 (0.59)	0 (0.00)	2 (1.32)
Males	24 (0.40)	7 (0.44)	11 (1.37)	3 (0.59)	5 (1.84)	5 (1.71)
Alcohol misuse	130 (1.04)	39 (1.33)	30 (2.04)	24 (2.82)	8 (1.79)	14 (3.13)
Females	73 (1.13)	17 (1.26)	14 (2.11)	12 (3.55)	5 (2.84)	3 (1.97)
Males	57 (0.95)	22 (1.39)	16 (1.99)	12 (2.34)	3 (1.10)	11 (3.72)
Non‐violent criminality	419 (3.37)	115 (3.93)	80 (5.45)	48 (5.64)	34 (7.59)	54 (12.50)
Females	118 (1.83)	34 (2.53)	17 (2.56)	15 (4.44)	8 (4.55)	10 (6.58)
Males	301 (5.01)	81 (5.12)	63 (7.84)	33 (6.43)	26 (9.56)	44 (14.86)
Violent criminality	462 (3.71)	122 (4.17)	86 (5.86)	55 (6.46)	35 (7.81)	58 (12.95)
Females	125 (1.94)	36 (2.67)	20 (3.01)	17 (5.03)	8 (4.55)	11 (7.24)
Males	337 (5.61)	86 (5.44)	66 (8.21)	38 (7.41)	27 (9.93)	47 (15.88)
No high school eligibility	494 (3.97)	164 (5.61)	88 (5.99)	71 (8.34)	45 (10.04)	57 (12.72)
Females	242 (6.14)	71 (8.96)	32 (8.23)	30 (16.48)	16 (18.8)	11 (16.92)
Males	252 (6.85	93 (9.66)	56 (11.43)	41 (13.85)	29 (19.08)	46 (28.05)
Social welfare recipiency	440 (3.0)	123 (4.21)	98 (6.68)	62 (7.29)	37 (8.26)	50 (11.16)
Females	265 (4.12)	63 (4.69)	45 (6.78)	31 (9.17)	10 (5.68)	9 (5.92)
Males	175 (2.91)	60 (3.80)	53 (6.59)	31 (6.04)	27 (9.93)	41 (13.85)

Table 2 shows that the sum of the conduct symptoms significantly predicted all outcomes, except for suicide among males. On the average, after statistically adjusting for maternal socioeconomic status, each additional conduct symptom was associated with a 31% increased risk of the adverse outcomes (mean hazard ratio = 1.32; range = 1.16, 1.56). Furthermore, to examine the dose–response relation, we split the conduct sum score into five categories (individuals with 1, 2, 3, 4, and 5–8 symptoms). Results supported a dose–response relation before and after adjusting for SES (Tables S1 and S3).

**Table 2:** Adverse outcomes in adolescence regressed on continuous conduct problems in childhood, adjusted for parental socioeconomic status

Adverse outcome	Females	Males
Depression	1.23 (1.12–1.38)	1.37 (1.23–1.53)
Anxiety	1.33 (1.20–1.46)	1.33 (1.22–1.48)
Antidepressant/sedative medication	1.25 (1.18–1.32)	1.34 (1.27–1.41)
Suicide attempt	1.30 (1.12–1.51)	1.16 (0.99–1.36)
Schizophrenia/ bipolar disorder	1.54 (1.22–1.93)	1.56 (1.22–1.93)
Substance abuse	1.33 (1.05–1.70)	1.34 (1.14–1.57)
Alcohol misuse	1.32 (1.15–1.50)	1.31 (1.17–1.47)
Nonviolent criminality	1.35 (1.22–1.49)	1.24 (1.18–1.31)
Violent criminality	1.35 (1.23–1.48)	1.22 (1.16–1.29)
No high school eligibility^a^	1.31 (1.21–1.42)	1.33 (1.26–1.41)
Social welfare recipiency^a^	1.20 (1.13–1.32)	1.38 (1.30–1.47)

Estimates represent hazard ratios (with 95% confidence intervals in parentheses).

a Estimates represent odds ratios based on logistic regression.

### Factor analyses of conduct symptoms and the adverse outcomes

For the eight parent‐reported conduct symptoms assessed in childhood, the a priori specified one‐factor model fit well for both boys and girls (Table S4). All symptoms had high loadings on the conduct problems factor among both boys (range = 0.68, 0.87) and girls (range = 0.62, 0.88; Table S4).

For the eleven adverse outcomes in emerging adulthood, the scree plot indicated the presence of two factors (Figure S1; Cattell, 1966). A two‐factor model fit well for both boys and girls (Table 3). The overlap between the two outcome factors was rotated toward one general comorbidity factor, as well as two independent factors that captured specific covariation among the outcomes above and beyond that explained by the general comorbidity factor (Waller, 2018). The outcomes loaded moderately to strongly on the general comorbidity factor; the first specific factor captured internalizing problems; and the second specific factor captured externalizing problems (Table 3). The factor loading patterns were highly similar after excluding antidepressant medication, high school ineligibility, and social welfare recipiency (Table S5).

**Table 3:** Standardized loadings on the adverse outcome factors in emerging adulthood by sex

Adverse outcomes	Males			Females		
	General factor	Specific int. factor	Specific ext. factor	General factor	Specific int. factor	Specific ext. factor
Depression	**0.56**	**0.69**	0.14	**0.55**	**0.72**	−0.17
Anxiety	**0.51**	**0.61**	−0.10	**0.53**	**0.64**	−0.11
Antidepressants/sedatives	**0.50**	**0.73**	−0.23	**0.53**	**0.70**	−0.17
Suicide attempt	0.29	0.16	0.13	**0.46**	**0.42**	0.04
Schizophrenia/ bipolar disorder	**0.55**	**0.48**	0.08	**0.61**	**0.48**	0.13
Drug abuse	**0.60**	0.02	**0.58**	**0.62**	**0.38**	0.24
Alcohol misuse	**0.45**	0.07	**0.38**	**0.46**	0.20	0.26
Nonviolent crimes	**0.38**	−0.18	**0.56**	**0.50**	−0.12	**0.62**
Violent crimes	**0.47**	−0.16	**0.63**	**0.51**	−0.24	**0.76**
High school ineligibility	**0.40**	−0.04	**0.45**	**0.36**	0.08	0.28
Social welfare recipiency	**0.42**	0.03	**0.39**	**0.42**	0.05	**0.37**

Int = internalizing. Ext = externalizing. Loadings greater than |0.29| are bolded.

Male model fit: RMSEA = 0.02, 90% CI: 0.02–0.03; CFI = 0.94; χ^2^ = 676.56, *df* = 448.

Female model fit: RMSEA = 0.03, 90% CI: 0.02–0.03; CFI = 0.96; χ^2^ = 704.92, *df* = 448.

### General and specific adverse outcome factors regressed onto the conduct problems factor

As displayed in Figure 2, the childhood conduct problems factor most strongly predicted the general comorbidity factor among boys (β = .45; 95% CI: 0.38, 0.51) and girls (β = .34; 95% CI: 0.26, 0.42) in emerging adulthood. To a lesser extent, the childhood conduct problems factor also significantly predicted the specific externalizing factor among boys (β = .22; 95% CI: 0.14, 0.29) and girls (β = .17; 95% CI: 0.08, 0.27) in emerging adulthood. Furthermore, above and beyond the influence on the general and externalizing factors, the childhood conduct problems factor also predicted the specific internalizing factor among both boys (β = .24; 95% CI: 0.18, 0.30) and girls (β = .17; 95% CI: 0.11, 0.23). The results were highly similar when we excluded antidepressant medication, high school ineligibility, and social welfare recipiency from the outcome factors (Figure S2).

### Twin analyses

**Figure 1:**
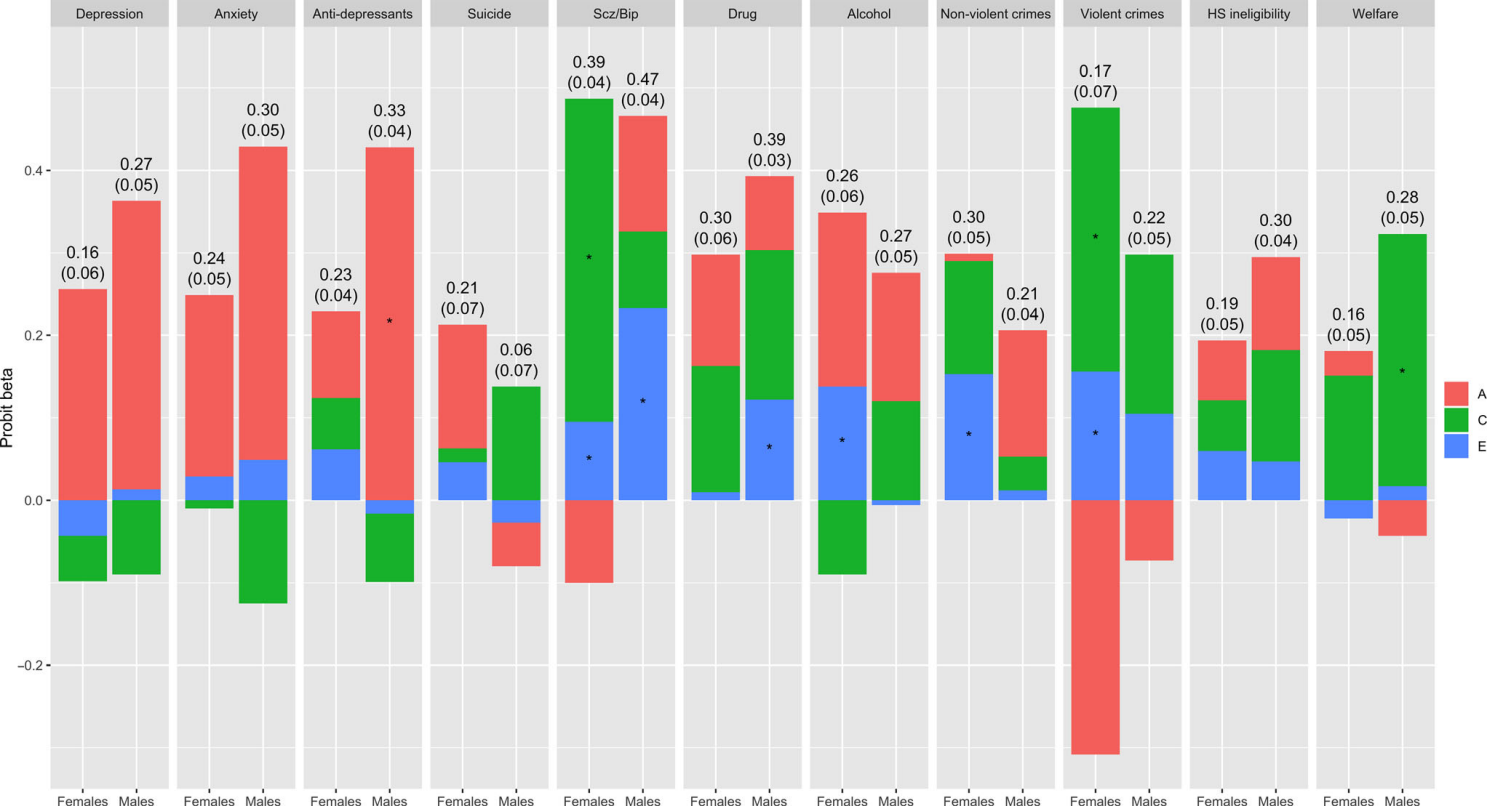
Adverse outcomes regressed on latent conduct problems. The observed (phenotypic) probit betas are presented on top of the bar charts (with standard errors in parentheses). The bar charts display the contributions of genetic (A), the shared environment (C), and the nonshared environment (E) to the observed probit betas. Scz/bip = Schizophrenia and/or bipolar disorder. HS = high school ineligibility. * = significant at *p* < .05 (see Table S6 for biometric estimates and standard errors)

The genetic, shared environmental, and nonshared environmental contributions to the phenotypic associations between conduct problems and each adverse outcome are displayed in Figure 1 (see Table S6 for estimates and standard errors). There was a trend for genetics to contribute primarily to the associations between conduct problems in childhood and depression, anxiety, and antidepressants in emerging adulthood. In contrast, the shared environment tended to contribute more the associations between conduct problems and violent criminality, drug abuse, and social welfare recipiency. Furthermore, the nonshared environment contributed significantly to the associations between conduct problems and schizophrenia/bipolar disorder, alcohol misuse, and nonviolent and violent criminality among females, and to schizophrenia/bipolar disorder and drug abuse among males.

**Figure 2:**
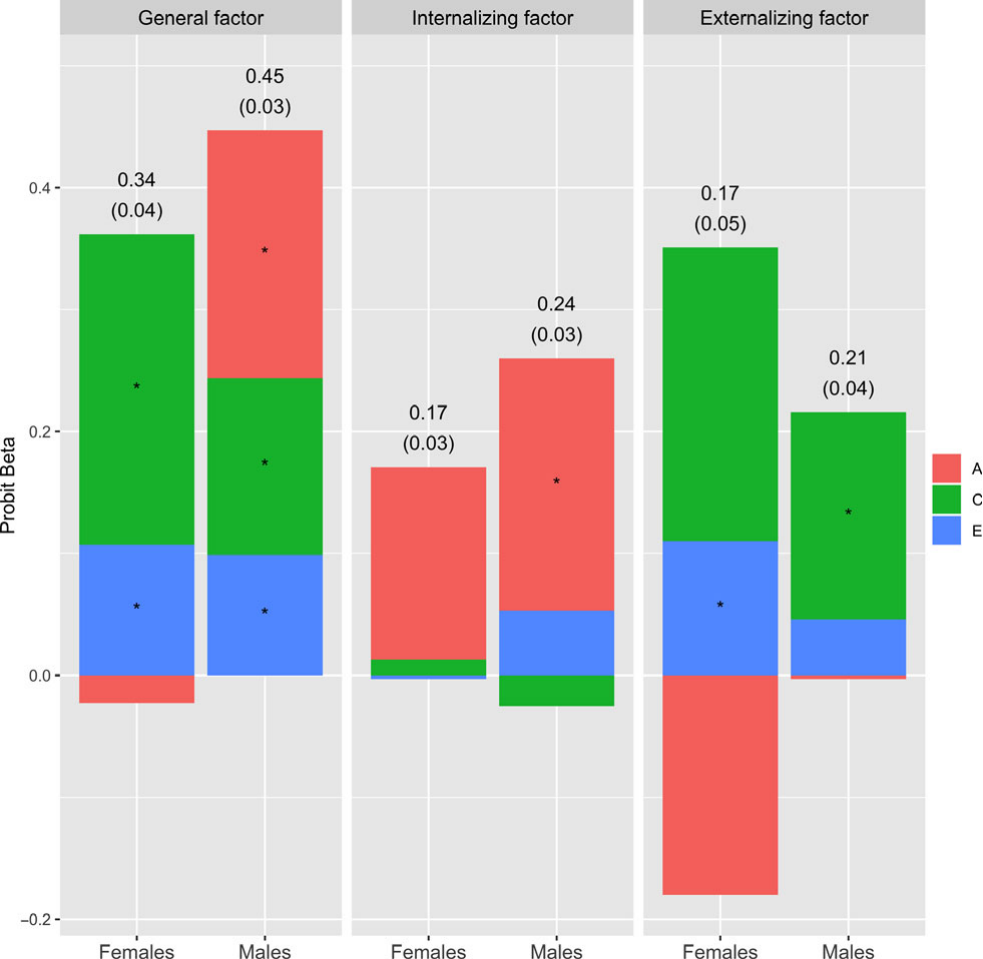
Latent general, internalizing, and externalizing factors regressed on latent conduct problems. The observed (phenotypic) probit betas are presented on top of the bar charts (with standard errors in parentheses). The bar charts display the contributions of genetic (A), the shared environment (C), and the nonshared environment (E) to the observed probit betas. * = significant at *p *< .05 (see Table S7 for biometric estimates and standard errors)

The genetic, shared environmental, and nonshared environmental contributions to associations between conduct problems and the general, internalizing, and externalizing factors are displayed in Figure 2 (see Table S7 for estimates and standard errors). The shared environment contributed substantially to the associations between childhood conduct problems and the general and specific externalizing factors in emerging adulthood. Shared genetics, on the other hand, contributed primarily to the association between childhood conduct problems and the internalizing factor in emerging adulthood. The nonshared environment contributed marginally but significantly to the association with the general factor for both men and women, and to the association with the externalizing factor among females. The biometric estimates were similar but the standard errors were slightly larger when we excluded antidepressant medication, high school ineligibility, and social welfare recipiency from the outcome factors (Table S8).

## Discussion

In this prospective cohort study based on a large, nationally representative sample of Swedish twins, childhood conduct problems were associated with subsequent externalizing outcomes, such as criminality and substance abuse. In addition, similar to previous smaller studies, we found associations of similar magnitude with later internalizing problems (Fergusson et al., 2005; Kim‐Cohen et al., 2003). Furthermore, the associations with the internalizing outcomes remained even after adjusting for later comorbidity and externalizing problems. Lastly, twin analyses demonstrated that the associations between childhood conduct problems and the externalizing adverse outcomes had a primarily shared environmental etiology, whereas genetics contributed primarily to the association between childhood conduct problems and the internalizing outcomes.

### Clinical implications

Emerging adulthood is characterized by the transition from dependence to independence, and this vulnerable period could be a 'tipping point' for lifelong mental and psychosocial problems (Arnett, 2007). These results suggest that childhood conduct problems might predict a particularly poor outlook in emerging adulthood. Therefore, it might be important for general child health care practitioners to assess childhood conduct problems for prognostic purposes, as these children appear to be at excess risk for not only later externalizing but also for later internalizing problems.

The different etiologies for the associations with the externalizing and internalizing outcomes might guide treatments toward familial versus individuals levels, respectively. The more pronounced influence of shared environmental factors on the association between childhood conduct problems and both the general comorbidity and the externalizing factors suggests that it might be important for treatments to target variables that are shared among siblings (e.g., parenting styles, familial financial resources, shared friends). Furthermore, we observed that several associations tended to persist within identical twin pairs. This might imply that interventions designed toward childhood conduct problems (e.g., the collaborative problem‐solving approach; Greene & Ablon, 2006) might alleviate a host of adverse outcomes (e.g., substance abuse, depression, or criminality) in emerging adulthood (D'Onofrio et al., 2013; Lahey & D'Onofrio, 2010; Turkheimer & Harden, 2014) or that the associations are a function of nonshared factors influencing one twin but not the other (e.g., a head injury to only one twin).

### Sex differences

Although there were large similarities between men and women, there were also some notable differences. First, in line with past research, men displayed a higher degree of externalizing problems, whereas women suffered from more internalizing problems (Kessler, Chiu, Demler, Merikangas, & Walters, 2005). Second, the associations between conduct problems in childhood and the adverse outcomes in emerging adulthood tended to be stronger among males. Third, among males, there was a trend for the shared environment to play a more significant role in the association between childhood conduct problems and the externalizing factor. Kendler and colleagues have speculated that adolescent men might be more susceptible to engage in reckless behaviors due to peer pressure, a shared environment component (Kendler et al., 2016).

### Implications for psychiatric nosology

Recent research has indicated that a general factor of psychopathology accounts for a substantial amount of the comorbidity among mental health problems (Caspi & Moffitt, 2018; Lahey et al., 2017). We replicated these results in a large sample of individuals entering adulthood based on a wide array of adverse outcomes drawn from national registers, including not only psychiatric diagnoses but also psychosocial outcomes (e.g., high school ineligibility). Furthermore, our results showed that childhood conduct problems were associated with the adversities in emerging adulthood primarily via the general factor, suggesting that the general comorbidity factor might be a useful nosological construct.

### Limitations

First, we studied twins, which might limit generalizability to singletons. Second, it is possible that some parents underreported conduct problems in their children, such that the risk estimates observed in our cohort might be on the conservative side of the true population effects. Third, the findings from our study need replication in another data set, using other measurements of externalizing and internalizing outcomes. Fourth, for the multivariate and twin analyses, we did not model the trajectory of the probability of the outcome (i.e., we ignored the influence of time), and instead only modeled whether the outcome had occurred or not. Finally, the response rate in the baseline assessment was 71%, and a validation study suggests that the nonresponders had more psychiatric and psychosocial problems than the responders did. Although we incorporated weights to adjust for the unequal probability of responding, it is possible that attrition could have biased the observed risk estimates to some extent.

## Conclusion

Relying on a large Swedish twin cohort, conduct problems in childhood uniquely predicted internalizing problems in emerging adulthood, above and beyond its associations with general and externalizing factors. It seems important to monitor boys and girls with conduct problems not only for future externalizing problems, but also for future internalizing problems. Treatment of specific outcomes, however, might require interventions at different levels.

## Supporting information


**Appendix S1**. Decomposing observed (phenotypic) regression beta into that which can be attributed to genetics, the shared environment, and the nonshared environment.
**Table S1**. Hazard ratios (HR) and corresponding 95% confidence intervals (CI) expressing associations between categorized childhood conduct symptoms and adverse outcomes in emerging adulthood, adjusted for socio‐economic status.
**Table S2**. Prevalences of conduct symptoms and adverse outcomes.
**Table S3**. Hazard ratios (HR) and corresponding 95% confidence intervals (CI) expressing associations between categorized childhood conduct symptoms and adverse outcomes in emerging adulthood.
**Table S4**. Standardized loadings on the childhood conduct factor by sex.
**Table S5**. Standardized loadings on the adverse outcomes factors in emerging adulthood by sex (excluding antidepressant medication, high school ineligibility, and social welfare recipiency).
**Table S6**. Contribution of genetics, the shared environment, and the nonshared environment to the phenotypic regression betas between conduct problems in childhood and observed adverse outcomes in emerging adulthood.
**Table S7**. Contribution of genetics, the shared environment, and the nonshared environment to the phenotypic regression betas between conduct problems in childhood and the general, externalizing, and internalizing factors in emerging adulthood.
**Table S8**. Contribution of genetics, the shared environment, and the nonshared environment to the phenotypic regression betas between conduct problems in childhood and the general, externalizing, and internalizing factors in emerging adulthood (excluding antidepressant medication, high school ineligibility, and social welfare recipiency).
**Figure S1**. Scree plot of the adverse outcomes by sex.
**Figure S2**. Latent general, internalizing, and externalizing factors regressed on latent conduct problems (excluding antidepressant medication, high school ineligibility, and social welfare recipiency).
