# Supplementary material for: Associations between conduct problems in childhood and adverse outcomes in emerging adulthood: a longitudinal Swedish nationwide twin cohort
Source: J Child Psychol Psychiatry. 2019 Dec 18;61(7):798–806. doi: 10.1111/jcpp.13169 (PMC7384167; doi:10.1111/jcpp.13169)
Supplement: Supplementary file 1 — Appendix S1. Decomposing observed (phenotypic) regression beta into that which can be attributed to genetics, the shared environment, and the nonshared environment. Table S1. Hazard ratios (HR) and corresponding 95% confidence intervals (CI) expressing associations between categorized childhood conduct symptoms and adverse outcomes in emerging adulthood, adjusted for socio‐economic status. Table S2. Prevalences of conduct symptoms and adverse outcomes. Table S3. Hazard ratios (HR) and corresponding 95% confidence intervals (CI) expressing associations between categorized childhood conduct symptoms and adverse outcomes in emerging adulthood. Table S4. Standardized loadings on the childhood conduct factor by sex. Table S5. Standardized loadings on the adverse outcomes factors in emerging adulthood by sex (excluding antidepressant medication, high school ineligibility, and social welfare recipiency). Table S6. Contribution of genetics, the shared environment, and the nonshared environment to the phenotypic regression betas between conduct problems in childhood and observed adverse outcomes in emerging adulthood. Table S7. Contribution of genetics, the shared environment, and the nonshared environment to the phenotypic regression betas between conduct problems in childhood and the general, externalizing, and internalizing factors in emerging adulthood. Table S8. Contribution of genetics, the shared environment, and the nonshared environment to the phenotypic regression betas between conduct problems in childhood and the general, externalizing, and internalizing factors in emerging adulthood (excluding antidepressant medication, high school ineligibility, and social welfare recipiency). Figure S1. Scree plot of the adverse outcomes by sex. Figure S2. Latent general, internalizing, and externalizing factors regressed on latent conduct problems (excluding antidepressant medication, high school ineligibility, and social welfare recipiency). [file JCPP-61-798-s001.docx]

**Online Supporting Information**

**Appendix S1.** Decomposing observed (phenotypic) regression beta into that which can be attributed to genetics, the shared environment, and the non-shared environment.

**Table S1.** Hazard ratios (HR) and corresponding 95% confidence intervals (CI) expressing associations between categorized childhood conduct symptoms and adverse outcomes in emerging adulthood, adjusted for socio-economic status.

**Table S2.** Prevalences of conduct symptoms and adverse outcomes.

**Table S3.** Hazard ratios (HR) and corresponding 95% confidence intervals (CI) expressing associations between categorized childhood conduct symptoms and adverse outcomes in emerging adulthood.

**Table S4.** Standardized loadings on the childhood conduct factor by sex.

**Table S5.** Standardized loadings on the adverse outcomes factors in emerging adulthood by sex (excluding anti-depressant medication, high school ineligibility, and social welfare recipiency).

**Table S6.** Contribution of genetics, the shared environment, and the non-shared environment to the phenotypic regression betas between conduct problems in childhood and observed adverse outcomes in emerging adulthood.

**Table S7.** Contribution of genetics, the shared environment, and the non-shared environment to the phenotypic regression betas between conduct problems in childhood and the general, externalizing, and internalizing factors in emerging adulthood.

**Table S8.** Contribution of genetics, the shared environment, and the non-shared environment to the phenotypic regression betas between conduct problems in childhood and the general, externalizing, and internalizing factors in emerging adulthood (excluding anti-depressant medication, high school ineligibility, and social welfare recipiency).

**Figure S1.** Scree plot of the adverse outcomes by sex.

**Figure S2.** Latent general, internalizing, and externalizing factors regressed on latent conduct problems (excluding anti-depressant medication, high school ineligibility, and social welfare recipiency).

**Appendix S1: Decomposing observed (phenotypic) regression beta into that which can be attributed to genetics, the shared environment, and the non-shared environment**

Let *R* be a covariance matrix between two variables, such that

$R=\left[\begin{matrix} r_{11} & r_{21} \\ r_{12} & r_{22} \end{matrix}\right]$,

where *r*_11_ is the variance for variable one, *r*_22_ is the variance for variable two, and *r*_12_ and *r*_21_ is the covariance between the two variables. Then *R* can be decomposed such that *R* = AA^T^ + CC^T^ + EE^T^, where

$$A=\left[\begin{matrix} a_{11} & 0 \\ a_{12} & a_{22} \end{matrix}\right],C=\left[\begin{matrix} c_{11} & 0 \\ c_{12} & c_{22} \end{matrix}\right],E=\left[\begin{matrix} e_{11} & 0 \\ e_{12} & e_{22} \end{matrix}\right],$$

and a_11_ is the genetic influence on the first variable, a_22_ is the genetic influence on the second variable, and a_12_ is the influence of the genetic part of the first variable onto the second variable.^32^ The same definitions apply to the C and E matrices. The observed regression beta between the two variables is then

$$\beta=\frac{a_{11}*a_{12}+c_{11}*c_{12}+e_{11}*e_{12}}{a^{2}_{11}+c^{2}_{11}+e^{2}_{11}}.$$

The relative contribution of genes to the observed beta is

$$\beta_{a}=\frac{a_{11}*a_{12}}{a^{2}_{11}+c^{2}_{11}+e^{2}_{11}},$$

and exchanging the numerator to c_11_*c_12_ or e_11_*e_12_ estimates the contribution of the shared ($\beta_{c}$) and non-shared environment ($\beta_{e}$), respectively, to the observed beta.

| Table S1. Hazard ratios (HR) and corresponding 95% confidence intervals (CI) expressing associations between childhood conduct symptoms and adverse outcomes in emerging adulthood, adjusted for socio-economic status. | | | | | | | | | | | |
| --- | --- | --- | --- | --- | --- | --- | --- | --- | --- | --- | --- |
|  | | Childhood conduct symptoms | | | | | | | | | |
|  | | 1 vs 0 | | 2 vs 0 | | 3 vs 0 | | 4 vs 0 | | 5-8 vs 0 | |
|  | | HR | CI | HR | CI | HR | CI | HR | CI | HR | CI |
| Depression | | 1.29 | 0.97-1.72 | 1.79 | 1.28-2.50 | 2.01 | 1.34-3.00 | 1.84 | 1.05-3.23 | 3.17 | 2.04-4.92 |
| Females | | 1.35 | 0.95-1.91 | 1.50 | 0.96-2.35 | 1.77 | 1.01-3.13 | 1.88 | 0.88-4-02 | 3.26 | 1.72-6.2 |
| Males | | 1.36 | 0.82-2.27 | 2.66 | 1.59-4.42 | 2.93 | 1.63-5.26 | 2.35 | 1.01-5.46 | 4.35 | 2.33-8.11 |
| Anxiety | | 0.99 | 0.74-1.31 | 1.84 | 1.38-2.47 | 2.70 | 1.97-3.69 | 2.24 | 1.42-3.53 | 3.40 | 2.33-4.97 |
| Females | | 0.79 | 0.53-1.18 | 1.91 | 1.32-2.78 | 2.91 | 1.91-4.44 | 2.05 | 1.05-4.02 | 4.16 | 2.45-7.05 |
| Males | | 1.39 | 0.92-2.10 | 1.93 | 1.21-3.09 | 2.92 | 1.91-4.70 | 2.87 | 1.53-5.38 | 3.61 | 2.08-6.24 |
| Anti-depressant/  sedative medication | | 1.31 | 1.14-1.51 | 1.68 | 1.42-1.98 | 2.31 | 1.92-2.78 | 2.63 | 2.07-3.33 | 4.38 | 3.59-5.33 |
| Females | | 1.29 | 1.08-1.55 | 1.47 | 1.17-1.86 | 2.09 | 1.58-2.76 | 2.13 | 1.46-3.11 | 4.13 | 3.00-5.70 |
| Males | | 1.43 | 1.16-1.76 | 2.07 | 1.63-2.63 | 2.82 | 2.19-3.65 | 3.47 | 2.54-4.73 | 5.34 | 4.13-6.91 |
| Suicide attempt | | 1.21 | 0.83-1.78 | 1.48 | 0.93-2.36 | 1.46 | 0.81-2.64 | 1.63 | 0.76-3.49 | 2.12 | 1.07-4.17 |
| Females | | 1.21 | 0.71-2.06 | 1.60 | 0.84-3.02 | 1.72 | 0.74-3.96 | 1.67 | 0.53-5.32 | 3.97 | 1.72-9.16 |
| Males | | 1.23 | 0.71-2.12 | 1.40 | 0.71-2.76 | 1.31 | 0.56-3.06 | 1.66 | 0.60-4.59 | 1.15 | 0.36-3.68 |
| Severe mental illness | | 1.11 | 0.72-2.72 | 2.19 | 0.89-5.37 | 4.44 | 1.90-10.34 | 4.86 | 1.68-14.06 | 7.38 | 3.00-18.12 |
| Females | | 0.95 | 0.27-3.27 | 1.92 | 0.56-6.63 | 5.11 | 1.70-15.42 | 2.51 | 0.33-18.99 | 9.06 | 2.62-31.31 |
| Males | | 1.43 | 0.38-5.37 | 2.77 | 0.74-10.46 | 4.36 | 1.16-16.43 | 8.27 | 2.19-31.17 | 7.63 | 2.03-28.78 |
| Substance abuse | | 1.26 | 0.68-2.34 | 3.06 | 1.73-5.42 | 1.65 | 0.66-4.17 | 3.21 | 1.27-8.08 | 4.51 | 2.03-10.01 |
| Females | | 1.44 | 0.58-3.58 | 2.42 | 0.91-6.44 | 1.91 | 0.44-8.20 | n/a | n/a | 4.54 | 1.06-19.50 |
| Males | | 1.11 | 0.48-2.58 | 3.38 | 1.66-6.90 | 1.44 | 0.44-4.81 | 4.59 | 1.75-12.03 | 4.22 | 1.61-11.06 |
| Alcohol misuse | 1.28 | | 0.89-1.83 | 1.95 | 1.31-2.90 | 2.71 | 1.76-4.20 | 1.72 | 0.84-3.51 | 3.03 | 1.75-5.26 |
| Females | 1.11 | | 0.66-1.89 | 1.86 | 1.05-3.30 | 3.19 | 1.73-5.87 | 2.60 | 1.05-6.43 | 1.81 | 0.57-5.76 |
| Males | 1.47 | | 0.90-2.40 | 2.08 | 1.19-3.62 | 2.46 | 1.32-4.59 | 1.14 | 0.36-3.65 | 3.89 | 2.04-7.42 |
| Non-violent criminality | 1.17 | | 0.95-1.44 | 1.63 | 1.28-2.07 | 1.69 | 1.26-2.28 | 2.32 | 1.64-3.29 | 3.84 | 2.90-5.11 |
| Females | 1.38 | | 0.94-2.03 | 1.40 | 0.84-2.33 | 2.48 | 1.45-2.43 | 2.60 | 1.27-5.31 | 3.83 | 2.01-7.31 |
| Males | 1.03 | | 0.88-1.30 | 1,57 | 1.20-2.03 | 1.28 | 0.89-1.84 | 1.94 | 1.30-2.89 | 3.18 | 2.31-4.36 |
| Violent criminality | 1.13 | | 0.92-1.38 | 1.59 | 1.26-2.00 | 1.76 | 1.33-2.33 | 2.16 | 1.53-3.05 | 3.73 | 2.84-4.91 |
| Females | 1.38 | | 0.96-2.00 | 1.56 | 0.97-2.50 | 2.66 | 1.60-4.41 | 2.45 | 1.20-5.02 | 3.98 | 2.15-7.37 |
| Males | 0.97 | | 0.77-1.22 | 1.47 | 1.13-1.92 | 1.32 | 0.94-1.84 | 1.79 | 1.21-2.64 | 3.00 | 2.22-4.08 |
| No  high school eligibility | 1.49* | | 1.24-1.80 | 1.61* | 1.27-2.04 | 2.52* | 1.93-3.29 | 3.33* | 2.38-4.66 | 4.78* | 3.50-6.54 |
| Females | 1,51* | | 1.14-1.99 | 1.37* | 0.93-2.01 | 3.02 | 2.00-4.56 | 3.40* | 1.95-5.93 | 3.11* | 1.61-6.04 |
| Males | 1.45* | | 1.13-1.87 | 1.76* | 1.29-2.38 | 2.19* | 1.54-3.11 | 3.21* | 2.10-4.90 | 5.30* | 3.69-7.63 |
| Social welfare recipiency | 1.19 | | 0.98-1.46 | 1.90 | 1.53-2.37 | 2.10 | 1.61-2.74 | 2.74 | 1.75-3.42 | 3.31 | 2.47-4.43 |
| Females | 1.17 | | 0.89-1.53 | 1.65 | 1.21-2.27 | 2.32 | 1.60-3.37 | 1.46 | 0.78-2.75 | 1.51 | 0.78-2.94 |
| Males | 1.31 | | 0.98-1.76 | 2.28 | 1.68-3.10 | 2.09 | 1.42-3.06 | 3.54 | 2.36-5.31 | 4.97 | 3.53-6.98 |
| *Note.* *logistic regression. | | | | | | | | | | | |

| Table S2. Prevalences of conduct symptoms and adverse outcomes. | | |
| --- | --- | --- |
| Childhood  conduct symptom count | Males (%) | Females (%) |
| 0 | 59.7 | 67.5 |
| 1 | 18.4 | 16.4 |
| 2 | 9.3 | 8.1 |
| 3 | 5.9 | 4.1 |
| 4 | 3.1 | 2.1 |
| 5-8 | 3.4 | 1.8 |
|  | | |
| Adverse mental health outcomes in emerging adulthood^1^ | Males (%) | Females (%) |
| Depression | 1.9 | 3.7 |
| Anxiety | 2.6 | 4.2 |
| Anti-depressants/sedatives | 9.6 | 13.6 |
| Suicide attempt | 1.8 | 2.1 |
| Schizophrenia/bipolar disorder | 0.4 | 0.5 |
| Drug abuse | 0.9 | 0.7 |
| Alcohol misuse | 1.9 | 2.1 |
| Non-violent crimes | 9.1 | 3.6 |
| Violent crimes | 2.7 | 0.7 |
| No high school eligibility | 8.7 | 7.2 |
| Social welfare recipiency | 6.1 | 7.2 |
| *Note.* ^1^Prevalences among those born before 1998 | | |

| Table S3. Hazard ratios (HR) and corresponding 95% confidence intervals (CI) expressing associations between childhood conduct symptoms and adverse outcomes in emerging adulthood. | | | | | | | | | | |
| --- | --- | --- | --- | --- | --- | --- | --- | --- | --- | --- |
|  | Childhood conduct symptoms | | | | | | | | | |
|  | 1 vs 0 | | 2 vs 0 | | 3 vs 0 | | 4 vs 0 | | 5-8 vs 0 | |
|  | HR | CI | HR | CI | HR | CI | HR | CI | HR | CI |
| Depression | 1.29 | 0.97-1.72 | 1.79 | 1.28-2.50 | 2.01 | 1.34-3.00 | 1.84 | 1.05-3.23 | 3.17 | 2.04-4.92 |
| Females | 1.35 | 0.95-1.91 | 1.50 | 0.96-2.35 | 1.77 | 1.01-3.13 | 1.88 | 0.88-4-02 | 3.26 | 1.72-6.2 |
| Males | 1.36 | 0.82-2.27 | 2.66 | 1.59-4.42 | 2.93 | 1.63-5.26 | 2.35 | 1.01-5.46 | 4.35 | 2.33-8.11 |
| Anxiety | 0.99 | 0.74-1.31 | 1.84 | 1.38-2.47 | 2.70 | 1.97-3.69 | 2.24 | 1.42-3.53 | 3.40 | 2.33-4.97 |
| Females | 0.79 | 0.53-1.18 | 1.91 | 1.32-2.78 | 2.91 | 1.91-4.44 | 2.05 | 1.05-4.02 | 4.16 | 2.45-7.05 |
| Males | 1.39 | 0.92-2.10 | 1.93 | 1.21-3.09 | 2.92 | 1.91-4.70 | 2.87 | 1.53-5.38 | 3.61 | 2.08-6.24 |
| Anti-depressant/  sedative medication | 1.31 | 1.14-1.51 | 1.68 | 1.42-1.98 | 2.31 | 1.92-2.78 | 2.63 | 2.07-3.33 | 4.38 | 3.59-5.33 |
| Females | 1.29 | 1.08-1.55 | 1.47 | 1.17-1.86 | 2.09 | 1.58-2.76 | 2.13 | 1.46-3.11 | 4.13 | 3.00-5.70 |
| Males | 1.43 | 1.16-1.76 | 2.07 | 1.63-2.63 | 2.82 | 2.19-3.65 | 3.47 | 2.54-4.73 | 5.34 | 4.13-6.91 |
| Suicide attempt | 1.21 | 0.83-1.78 | 1.48 | 0.93-2.36 | 1.46 | 0.81-2.64 | 1.63 | 0.76-3.49 | 2.12 | 1.07-4.17 |
| Females | 1.21 | 0.71-2.06 | 1.60 | 0.84-3.02 | 1.72 | 0.74-3.96 | 1.67 | 0.53-5.32 | 3.97 | 1.72-9.16 |
| Males | 1.23 | 0.71-2.12 | 1.40 | 0.71-2.76 | 1.31 | 0.56-3.06 | 1.66 | 0.60-4.59 | 1.15 | 0.36-3.68 |
| Severe mental illness | 1.11 | 0.72-2.72 | 2.19 | 0.89-5.37 | 4.44 | 1.90-10.34 | 4.86 | 1.68-14.06 | 7.38 | 3.00-18.12 |
| Females | 0.95 | 0.27-3.27 | 1.92 | 0.56-6.63 | 5.11 | 1.70-15.42 | 2.51 | 0.33-18.99 | 9.06 | 2.62-31.31 |
| Males | 1.43 | 0.38-5.37 | 2.77 | 0.74-10.46 | 4.36 | 1.16-16.43 | 8.27 | 2.19-31.17 | 7.63 | 2.03-28.78 |
| Substance abuse | 1.26 | 0.68-2.34 | 3.06 | 1.73-5.42 | 1.65 | 0.66-4.17 | 3.21 | 1.27-8.08 | 4.51 | 2.03-10.01 |
| Females | 1.44 | 0.58-3.58 | 2.42 | 0.91-6.44 | 1.91 | 0.44-8.20 | n/a | n/a | 4.54 | 1.06-19.50 |
| Males | 1.11 | 0.48-2.58 | 3.38 | 1.66-6.90 | 1.44 | 0.44-4.81 | 4.59 | 1.75-12.03 | 4.22 | 1.61-11.06 |
| Alcohol misuse | 1.28 | 0.89-1.83 | 1.95 | 1.31-2.90 | 2.71 | 1.76-4.20 | 1.72 | 0.84-3.51 | 3.03 | 1.75-5.26 |
| Females | 1.11 | 0.66-1.89 | 1.86 | 1.05-3.30 | 3.19 | 1.73-5.87 | 2.60 | 1.05-6.43 | 1.81 | 0.57-5.76 |
| Males | 1.47 | 0.90-2.40 | 2.08 | 1.19-3.62 | 2.46 | 1.32-4.59 | 1.14 | 0.36-3.65 | 3.89 | 2.04-7.42 |
| Non-violent criminality | 1.17 | 0.95-1.44 | 1.63 | 1.28-2.07 | 1.69 | 1.26-2.28 | 2.32 | 1.64-3.29 | 3.84 | 2.90-5.11 |
| Females | 1.38 | 0.94-2.03 | 1.40 | 0.84-2.33 | 2.48 | 1.45-2.43 | 2.60 | 1.27-5.31 | 3.83 | 2.01-7.31 |
| Males | 1.03 | 0.88-1.30 | 1,57 | 1.20-2.03 | 1.28 | 0.89-1.84 | 1.94 | 1.30-2.89 | 3.18 | 2.31-4.36 |
| Violent criminality | 1.13 | 0.92-1.38 | 1.59 | 1.26-2.00 | 1.76 | 1.33-2.33 | 2.16 | 1.53-3.05 | 3.73 | 2.84-4.91 |
| Females | 1.38 | 0.96-2.00 | 1.56 | 0.97-2.50 | 2.66 | 1.60-4.41 | 2.45 | 1.20-5.02 | 3.98 | 2.15-7.37 |
| Males | 0.97 | 0.77-1.22 | 1.47 | 1.13-1.92 | 1.32 | 0.94-1.84 | 1.79 | 1.21-2.64 | 3.00 | 2.22-4.08 |
| No  high school eligibility | 1.49* | 1.24-1.80 | 1.61* | 1.27-2.04 | 2.52* | 1.93-3.29 | 3.33* | 2.38-4.66 | 4.78* | 3.50-6.54 |
| Females | 1.51* | 1.14-1.99 | 1.37* | 0.93-2.01 | 3.02 | 2.00-4.56 | 3.40* | 1.95-5.93 | 3.11* | 1.61-6.04 |
| Males | 1.45* | 1.13-1.87 | 1.76* | 1.29-2.38 | 2.19* | 1.54-3.11 | 3.21* | 2.10-4.90 | 5.30* | 3.69-7.63 |
| Social welfare recipiency | 1.19 | 0.98-1.46 | 1.90 | 1.53-2.37 | 2.10 | 1.61-2.74 | 2.74 | 1.75-3.42 | 3.31 | 2.47-4.43 |
| Females | 1.17 | 0.89-1.53 | 1.65 | 1.21-2.27 | 2.32 | 1.60-3.37 | 1.46 | 0.78-2.75 | 1.51 | 0.78-2.94 |
| Males | 1.31 | 0.98-1.76 | 2.28 | 1.68-3.10 | 2.09 | 1.42-3.06 | 3.54 | 2.36-5.31 | 4.97 | 3.53-6.98 |
| *Note.* *logistic regression. | | | | | | | | | | |

| Table S4. Standardized loadings on the childhood conduct problems factor by sex. | | |
| --- | --- | --- |
| Parent-rated childhood conduct symptoms | Males | Females |
| Has there ever been a time when s/he would be angry to the extent that s/he cannot be reached? | **0.74** | **0.62** |
| Does s/he often argue with adults? | **0.87** | **0.81** |
| Does s/he often tease others by deliberately doing things that are perceived as provocative? | **0.68** | **0.74** |
| Is s/he easily offended, or disturbed by others? | **0.82** | **0.83** |
| Is s/he easily teased? | **0.83** | **0.83** |
| Has s/he ever deliberately been physically cruel to anybody? | **0.79** | **0.85** |
| Does s/he often start fights? | **0.86** | **0.88** |
| Does s/he steal or destroy things at home or outside home? | **0.83** | **0.82** |
| *Notes*. Loadings greater than \|.29\| are bolded.  Male model fit: Root Mean Square Error of Approximation, RMSEA = .02, 90% CI: .01-.02; Confirmatory Fit Index, CFI = .99; χ^2^ = 357.33, degrees of freedom, *df* = 262.  Female model fit: RMSEA = 0.02, 90% CI: 0.02 - .03; CFI = 0.97; χ2 = 385.78, *df*, = 262. | | |

| Table S5. Standardized loadings on the adverse outcomes factors in emerging adulthood by sex (excluding anti-depressant medication, high school ineligibility, and social welfare recipiency). | | | | | | | |
| --- | --- | --- | --- | --- | --- | --- | --- |
|  | Males | | |  | Females | | |
| Adverse outcomes | General factor | Specific int. factor | Specific ext. factor |  | General factor | Specific int. factor | Specific ext. factor |
| Depression | **0.44** | **0.64** | -0.20 |  | **0.48** | **0.73** | -0.25 |
| Anxiety | **0.43** | **0.62** | -0.19 |  | **0.46** | **0.66** | -0.20 |
| Suicide attempt | 0.27 | 0.19 | 0.08 |  | **0.42** | **0.46** | -0.04 |
| Schizophrenia/ bipolar disorder | **0.56** | **0.64** | -0.08 |  | **0.62** | **0.57** | 0.05 |
| Drug abuse | **0.60** | 0.10 | **0.50** |  | **0.59** | **0.45** | 0.14 |
| Alcohol misuse | **0.44** | 0.03 | **0.41** |  | **0.46** | 0.27 | 0.19 |
| Non-violent crimes | **0.36** | -0.29 | **0.65** |  | **0.59** | 0.01 | **0.58** |
| Violent crimes | **0.46** | -0.23 | **0.69** |  | **0.63** | -0.12 | **0.75** |
| *Notes*. Int = internalizing. Ext = externalizing. Loadings greater than \|.29\| are bolded.  Male model fit: RMSEA = 0.02, 90% CI: 0.02 - .03; CFI = 0.93; χ2 = 353.69, *df*, = 229.  Female model fit: RMSEA = 0.03, 90% CI: 0.03 - .03; CFI = 0.93; χ2 = 404.64, *df*, = 229. | | | | | | | |

| Table S6. Contribution of genetics, the shared environment, and the non-shared environment to the phenotypic regression betas between conduct problems in childhood and observed adverse outcomes in emerging adulthood. | | | | | | | |
| --- | --- | --- | --- | --- | --- | --- | --- |
|  | Genetic, environmental, and non-shared environmental contributions | | | | | | |
|  | Males | | |  | Females | | |
| Outcomes | *β_a_* | *β_c_* | *β_e_* |  | *β_a_* | *β_c_* | *β_e_* |
| Depression | 0.350 (0.227) | -0.090 (0.160) | 0.013 (0.074) |  | 0.256  (0.236) | -0.055 (0.182) | -0.043  (0.077) |
| Anxiety | 0.380 (0.201) | -0.125 (0.143) | 0.049 (0.073) |  | 0.220  (0.211) | -0.010 (0.169) | -0.029 (0.071) |
| Anti-depressants/  sedatives | 0.428 (0.161) | -0.083 (0.115) | -0.016 (.052) |  | 0.105 (0.169) | 0.062 (0.135) | 0.062  (0.054) |
| Suicide | -0.053 (0.256) | 0.138 (0.207) | -0.027 (0.082) |  | 0.150 (0.283) | 0.017 (0.240) | 0.046  (0.082) |
| Schizophrenia/bipolar disorder | 0.140 (0.196) | 0.093 (0.117) | 0.233 (0.087) |  | -0.100 (0.170) | 0.392 (0.137) | 0.095  (0.048) |
| Drug abuse | 0.090 (0.185) | 0.181 (0.169) | 0.122 (0.029) |  | 0.135 (0.203) | 0.153 (0.176) | 0.010  (0.074) |
| Alcohol misuse | 0.156 (0.193) | 0.120 (0.157) | -0.006 (0.066) |  | 0.211 (0.260) | -0.090 (0.204) | 0.138 (0.066) |
| Non-violent crimes | 0.153 (0.174) | 0.041 (0.124) | 0.012 (0.057) |  | 0.009 (0.235) | 0.137 (0.168) | 0.153 (0.077) |
| Violent crimes | -0.073 (0.230) | 0.193 (0.177) | 0.105 (0.079) |  | -0.308 (0.247) | 0.320 (0.181) | 0.156 (0.056) |
| High school ineligibility | 0.113 (0.157) | 0.135 (0.117) | 0.047 (0.051) |  | 0.073 (0.204) | 0.061 (0.156) | 0.060 (0.060) |
| Social welfare recipiency | -0.043 (0.203) | 0.306 (0.140) | 0.017 (0.064) |  | 0.030 (0.202) | 0.151 (0.147) | -0.022 (0.051) |
| *Notes*. Standardized regression probit betas with standard errors in parentheses.  *β_a_* = Genetic contribution to phenotypic beta.  *β_c_* = Shared environment contribution to phenotypic beta.  *β_e_* = Non-shared environment contribution to phenotypic beta. | | | | | | | |

| Table S7. Contribution of genetics, the shared environment, and the non-shared environment to the phenotypic regression betas between conduct problems in childhood and the general, externalizing, and internalizing factors in emerging adulthood. | | | | | | | |
| --- | --- | --- | --- | --- | --- | --- | --- |
|  | Genetic, environmental, and non-shared environmental contributions | | | | | | |
|  | Males | | |  | Females | | |
| Outcomes | *β_a_* | *β_c_* | *β_e_* |  | *β_a_* | *β_c_* | *β_e_* |
| General factor | 0.203 (0.093) | 0.145 (0.071) | 0.099 (0.035) |  | -0.023  (0.128) | 0.255 (0.101) | 0.107  (0.038) |
| Specific internalizing factor | 0.207 (0.047) | -0.025 (0.018) | 0.053 (0.031) |  | 0.158  (0.101) | 0.013 (0.087) | -0.003 (0.028) |
| Specific externalizing factor | -0.003 (0.093) | 0.170  (0.075) | 0.046  (0.031) |  | -0.180  (0.173) | 0.241  (0.141) | 0.110  (0.036) |
| *Notes*. Standardized regression betas with standard errors in parentheses.  *β_a_* = Genetic contribution to phenotypic beta.  *β_c_* = Shared environment contribution to phenotypic beta.  *β_e_* = Non-shared environment contribution to phenotypic beta. | | | | | | | |

| Table S8. Contribution of genetics, the shared environment, and the non-shared environment to the phenotypic regression betas between conduct problems in childhood and the general, externalizing, and internalizing factors in emerging adulthood (excluding anti-depressant medication, high school ineligibility, and social welfare recipiency). | | | | | | | |
| --- | --- | --- | --- | --- | --- | --- | --- |
|  | Genetic, environmental, and non-shared environmental contributions | | | | | | |
|  | Males | | |  | Females | | |
| Outcomes | *β_a_* | *β_c_* | *β_e_* |  | *β_a_* | *β_c_* | *β_e_* |
| General factor | 0.207 (0.119) | 0.115 (0.092) | 0.128 (0.036) |  | -0.015  (0.143) | 0.248 (0.114) | 0.099  (0.043) |
| Specific internalizing factor | 0.150 (0.122) | 0.009 (0.088) | 0.130 (0.047) |  | 0.203  (0.098) | 0.007 (0.086) | -0.023 (0.028) |
| Specific externalizing factor | 0.058 (0.114) | 0.106  (0.092) | -0.002  (0.030) |  | -0.219  (0.192) | 0.240  (0.160) | 0.122  (0.040) |
| *Notes*. Standardized regression betas with standard errors in parentheses.  *β_a_* = Genetic contribution to phenotypic beta.  *β_c_* = Shared environment contribution to phenotypic beta.  *β_e_* = Non-shared environment contribution to phenotypic beta. | | | | | | | |

**Figure S1.** Scree plot of the adverse outcomes by sex.


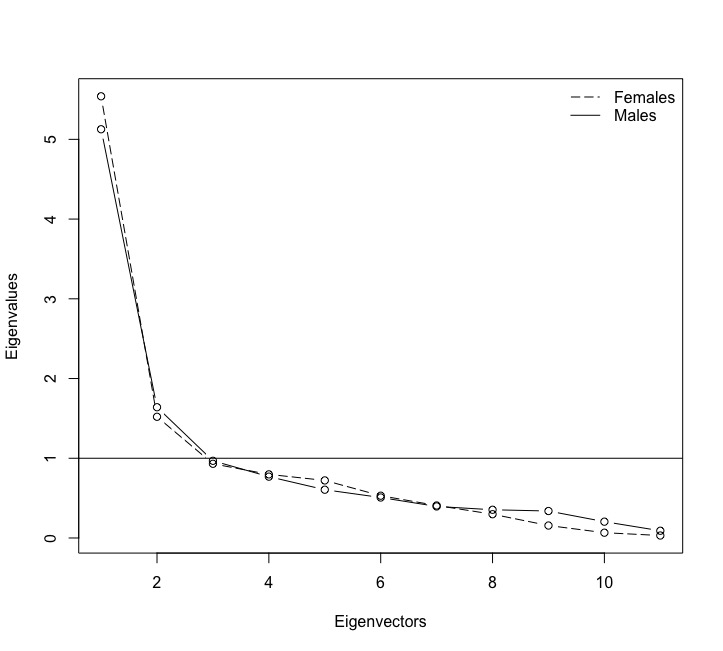


**Figure S2.** Latent general, internalizing, and externalizing factors regressed on latent conduct problems (excluding anti-depressant medication, high school ineligibility, and social welfare recipiency).


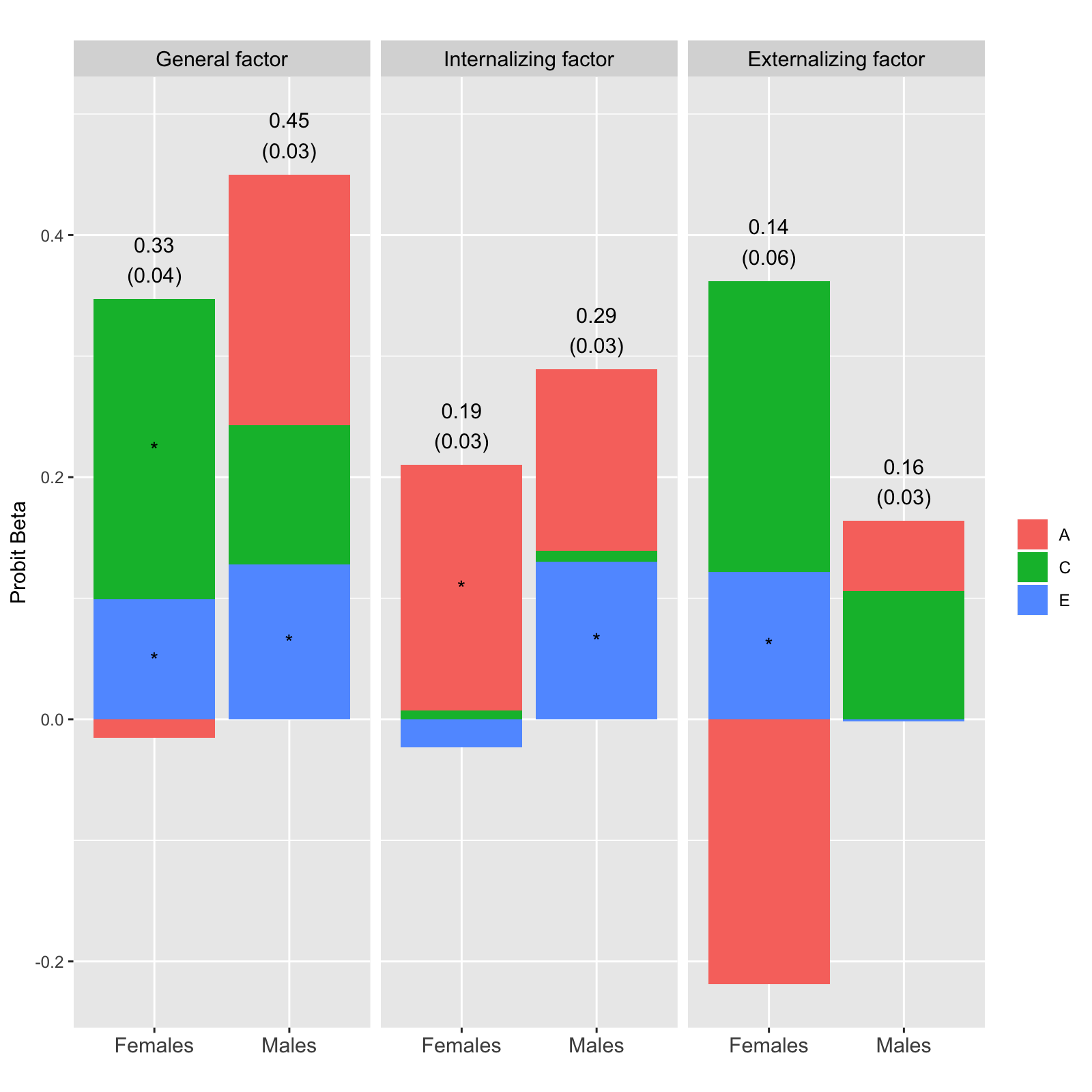


*Figure legend. The observed (phenotypic) probit betas are presented on top of the bar charts (with standard errors in parentheses). The bar charts display the contributions of genetic (A), the shared environment (C), and the non-shared environment (E) to the observed probit betas. * = significant at p < .05 (see eTable 8 for biometric estimates and standard errors).*
